# Studying caregiver-infant co-regulation in dynamic, diverse cultural contexts: A call to action

**DOI:** 10.1016/j.infbeh.2021.101586

**Published:** 2021-06-09

**Authors:** Andrea C. Buhler-Wassmann, Leah C. Hibel

**Affiliations:** University of California, Davis, United States

**Keywords:** Co-regulation, Caregiver-infant interactions, Culture, Context, Daily life

## Abstract

Caregivers and infants co-regulate their physiology, emotions, and behavior in a way that is dynamically responsive to each other and the contexts in which they live. This paper is an introduction and call to action for researchers interested in understanding how to study caregiver-infant interactions in the home and diverse cultural contexts, including marginalized communities. We argue that research will be more valid, culturally relevant, and tapped-in to the daily lives of caregivers and infants if there is partnership and collaboration with the caregivers in the design of the questions, data collection and analysis, and distribution of the findings. We recommend dynamically assessing emotions, behaviors, and physiology using repeated sampling methods including ecological momentary assessments (EMA), salivary bioscience, and actigraphy. We aim to extend current practices of studying caregiver-infant co-regulation by measuring fluctuations of daily life and considering sociocultural factors that shape naturalistic caregiver-infant interactions. Using methodological advancements and community-based participatory research approaches can enable developmental scientists to measure life as it is actually lived.

## 1. Introduction

Beginning in infancy, humans’ biological, emotional, and behavioral systems are organizing, integrating, and responding to their social environments (Darling & Steinberg, 2017; Posner & Rothbart, 2000; Tucker, Luu, & Pribram, 1995). Infants are reliant on their caregivers and have an evolutionary need to be cared for and loved in order to survive (Bowlby, 1969). Due to limited self-regulatory and communication capacities, infants require caregiver support to stabilize their emotions, physiology, and behavior when they are expressing negative affect, physiological arousal, or unmet needs (Beeghly & Tronick, 2011). Caregivers meet their infants’ needs by providing supportive, sensitive caregiving, which involves an accurate perception of the infant’s signals and prompt, appropriate responses (Ainsworth, Bell, & Stayton, 1974; Bowlby, 1969). Caregiver-child interactions exist within a dynamic, fast-paced, complex world where caregivers are simultaneously responding to their children as well as other social, financial, work, community, political, nutritional, and personal demands. Further, intergenerational transmission of cultural norms, behaviors, and values influences how and why caregivers respond to their child at any given moment. Accurate assessment of these complex, context dependent interactions necessitates methods that capture naturalistic fluctuations and life as it is lived, as well as collaborative and trusting relationships between families and researchers. Thus, this paper aims to provide researchers with the theoretical framework and methodological guidelines to 1) study the naturalistic process of caregiver-infant co-regulation as it occurs in real time, in the real world and 2) collaboratively engage families to ensure the assessments meaningfully attend to the features of their daily lives and center their experiences.

## 2. Theoretical and conceptual understanding of co-regulation

Caregiver-infant co-regulation is the bidirectional process of organizing physiology, emotion, and behaviors in response to caregiver-infant interactions (Butler & Randall, 2013). This process occurs day-by-day on a moment-to-moment basis. For example, infant hunger, pain, discomfort, sleepiness, or overstimulation might cause infant fussiness and distress (Hall, Levenson, & Hasler, 2012; Zeifman, 2001). Infant distress is a powerful elicitor of maternal behavior (Leerkes, 2010). In fact, crying is thought to have evolved to evoke maternal care (Bowlby, 1969, 1982; Out, Pieper, Bakermans-Kranenburg, Zeskind, & van IJzendoorn, 2010). Infants’ cues motivate parental responses, with infant crying prompting a range of caregiver reactions, from closeness (Ainsworth, 1979) to anger, anxiety, or withdrawal (Frodi & Lamb, 1980; Hibel, Buhler-Wassmann, Trumbell, & Liu, 2019; Leerkes, Parade, & Gudmundson, 2011). Likewise, caregiver emotion, behavior, and physiology are associated with differences in infant physiology (Hernández-Martínez et al., 2019; Hibel & Mercado, 2019; Tarullo, John, & Meyer, 2017). Caregivers’ responses serve to either up or down regulate infant distress (DePasquale, 2020; Gunnar, 2017). When caregivers engage in interactive activities such as playing, reading, feeding, bathing, changing/dressing, and holding, children develop greater capacities to be soothed, enjoyment derived from being held, and control of their attention and emotion arousal (Bridgett et al., 2011). On the other hand, when caregivers react with harsh and punitive or withdrawn and disengaged behaviors, infant self-soothing and flexible control of attention and arousal are diminished (Bridgett, Burt, Edwards, & Deater-Deckard, 2015). Through this dynamic process of infant destabilization and caregivers’ responses, infants’ interconnected biobehavioral systems develop (Montirosso & McGlone, 2020; Thelen & Smith, 2007).

Stress responsive physiological systems and restorative sleep physiology underlie emotions and behaviors, supporting infant and caregiver reactivity and regulation. One of the biological systems that is influenced by caregiver-infant interactions is the hypothalamic-pituitary-adrenal (HPA) axis, which responds to stress and regulates arousal, emotion, and mood, along with many bodily functions like digestion and the immune system (Hostinar, Sullivan, & Gunnar, 2014). The end product of the HPA axis is cortisol, which is produced in a daily circadian rhythm as well as in response to acute stressors (Gunnar & Adam, 2012). Diurnal cortisol supports daily functioning, can be measured through salivary assay, and follows a predictable pattern of high levels in the morning with a steep decline (i.e. slope) throughout the day (Charmandari, Tsigos, & Chrousos, 2005). The quality of the caregiver-infant relationship influences this system, with more secure attachment associated with healthier cortisol patterns and disorganized attachment contributing to flatter diurnal cortisol slopes (Bernard & Dozier, 2010; Cohen & Wills, 1985; Gunnar, Brodersen, Nachmias, Buss, & Rigatuso, 1996; Hibel, Marceau, & Buhler-Wassmann, 2020; Luijk et al., 2010).

Likewise, the autonomic nervous system (ANS) is also thought to dynamically co-regulate during caregiver-infant interactions (Feldman, 2007a, 2007b). The ANS is made up of the parasympathetic nervous system, which reduces physiological arousal, and the sympathetic nervous system, which is responsive to danger, fear, or challenge. Autonomic activation is indexed by a number of different ANS markers (e.g., heart rate, heart rate variability, salivary alpha amylase) which reflect different aspects of ANS activity (Ali & Nater, 2020). Most of these measures require laboratory-based equipment (e.g., heart rate variability, skin conductance) or blood or spinal fluid draws (e.g., epinephrine, norepinephrine); however, salivary alpha amylase, a reliable and valid measure of the ANS, can be collected non-invasively via saliva.

Baseline levels of ANS functioning and ANS reactivity represent caregivers’ and infants’ perceptions of security or threat in response to environmental stimuli or stressors (Alen et al., 2021). For example, separations between infants and their primary caregivers activate stress responses and changes in both the parasympathetic and sympathetic nervous systems. Specifically, infants’ heart rates increase when they are separated from their caregivers and decrease once caregivers reunite with their infants (Roder et al., 2020). Caregivers’ parasympathetic functioning influences both their parenting behaviors and infants’ parasympathetic system on a moment-to-moment basis (e.g., DePasquale, 2020). In sum, a caregiver’s responses to infant cues in part regulates the infant’s physiological arousal, creating a sense of calm and security in the infant which is thought to form the basis of the attachment relationship (Bowlby, 1969; Feldman, 2007a, 2007b).

Sleep in infancy is co-regulated by caregivers, who help decrease infants’ arousal through soothing interactions (El-Sheikh & Kelly, 2017; Feldman, 2012; Sadeh, Tikotzky, & Scher, 2010; Tikotzky et al., 2015). Insecure attachment relationships have been linked with more child bedtime problems (Benoit, Zeanah, Boucher, & Minde, 1992) whereas infants sleep the longest when mothers are more nurturing, display less close contact with infants at bedtime, and engage in less arousing bedtime activities (Philbrook & Teti, 2016). Regulatory systems are linked such that when infants sleep well, they are better able to regulate their emotions (Feldman et al., 2009; Mindell, Leichman, DuMond, & Sadeh, 2017). The effect of stressful experiences in everyday life, such as job loss, change in income, marital problems, illness, and new pregnancies, on infants’ wellbeing may be mediated by the impact that they have on caregivers’ emotions and behaviors (Fields, Harmon, Lee, Louie, & Tottenham, 2021). Specifically, parental anxiety in response to contextual stress has been associated with young children’s cortisol elevations and behavioral issues (Fields et al., 2021). Caregiver-infant interactions at bedtime and feeding also shape stress physiology. Infants who co-sleep, either in the same bed or the same room, with their caregivers and are breastfed during the first six months of life experienced lower cortisol reactivity and greater cortisol recovery, respectively, to the stress of caregiver-infant separation at 12 months old (Beijers, Riksen-Walraven, & de Weerth, 2013). Thus, cortisol, emotions, sleep, and stressful everyday experiences are important to study to understand caregiver-infant co-regulation in context.

As young children increase their regulatory capacities, they show increases in positive affect (Tronick, 1989) and physiological recovery to distress (Conradt & Ablow, 2010). Over time, how caregivers respond to infant cues sets the stage for long term regulatory abilities. Prompt, sensitive responses predict greater prosocial behavior and fewer internalizing and externalizing symptoms (Leerkes, Blankson, & O’Brien, 2009; Moed, Dix, Anderson, & Greene, 2017). Conversely, harsh, punitive, or withdrawn caregiving jeopardizes children’s ability to appropriately manage challenge, increasing internalizing and externalizing symptoms (Feldman et al., 2009; Harden, Buhler, & Parra, 2016; Margolin & Gordis, 2004). Co-regulation is the precursor to self-regulation, which is flexible regulation of emotional, behavioral, and biological systems (Bridgett et al., 2015). Children who have the ability to exercise control over their thoughts, feelings, and behaviors are more likely to be socially and academically competent and less likely to be anxious, depressed, aggressive, physically ill, unemployed, and use substances later in life (Robson, Allen, & Howard, 2020). This important process of regulating biological, emotional, and behavioral responses begins with caregiver-infant interactions in their home contexts (e.g., Bush & Peterson, 2013).

### 2.1. Co-regulation occurs within the family context

Caregivers’ and infants’ biology, emotion, and behavior fluctuate throughout the day depending on the features of daily life, including the family’s experiences (e.g., stressors and supports, emotional climate within the family, work schedules, social positioning) and their culture (García Coll et al., 1996; Repetti, Reynolds, & Sears, 2015; Thompson, 2019; Vélez-Agosto, Soto-Crespo, Vizcarrondo-Oppenheimer, Vega-Molina, & García Coll, 2017). Cultural practices refer to “behaviors that are learned, shared, and exhibited by a group of people” (Yosso, 2005, pg. 75). Culture impacts all aspects of family life, from the places infants sleep, to the activities infants experience, to how infant cues are interpreted, to how caregivers respond to infant cues (Bornstein et al., 2017; Campos & Kim, 2017; Kuchirko & Tamis-LeMonda, 2019; Vélez-Agosto et al., 2017). Understanding and accounting for family experiences and culture provides a more accurate depiction of caregiver-infant interactions as they occur in the dyad’s day to day life. In other words, assessing family life and culture provides necessary information on co-regulation as it is actually experienced by the infant. Thus, misrepresenting naturalistic caregiver-infant interactions does not allow for a scientific understanding of caregiver-infant co-regulation in response to the challenges they face on a daily basis and therefore leads to misrepresentation of the development of biological and emotion regulation. In order to assess and represent family experiences and culture in an ethical, culturally appropriate, and inclusive way that centers the families, caregivers must be engaged throughout the research process.

#### 2.1.1. Family experiences

Families experience a wide range of naturally occurring stressors and supports which influence caregivers’ and infants’ biobehavioral expressions. Infants oscillate between exploration, hunger, sleep, comfort, and discomfort and require caregivers’ attention, diaper changes, soothing, and feeding. Caregivers’ responses to infant demands are not just reflective of stable caregiving behaviors, but the dynamic pressures and supports a family experiences moment-to-moment, day-to-day, and year-to-year. For example, caregivers’ behavior and regulation of children’s states are sensitive to daily events, such as caregiver work schedules (Hibel, Trumbell, & Mercado, 2014), daily stress (Repetti & Wood, 1997), and interactions with other family members (Gordon, Zagoory-Sharon, Leckman, & Feldman, 2010; Jewell, Luecken, Gress-Smith, Crnic, & Gonzales, 2015). Caregivers who experience and perceive more daily hassles, life stressors, and less social support have more trouble sleeping and also engage in more dysfunctional parenting and less observed positive parenting (McQuillan, Bates, Staples, & Deater-Deckard, 2019). Similarly, parents who report being more exhausted during the week report lower quality parent-child relationship during the weekend (Gillis & Roskam, 2019). Altogether this body of work suggests that the amount of stress or emotional distress experienced during the day affects the caregivers’ and infants’ physiological arousal and sleep quality, impacting caregivers’ sensitivity and the quality of interactions. Given infants’ reliance on their caregivers, the caregivers’ and infants’ highs and lows are experienced together. Some moments are marked by synchrony (i.e., matched behavior, emotion, or physiology) and others are mismatched (DePasquale, 2020). It is an ever changing, dynamic, and sometimes chaotic life that includes the highest intensities of crying or fighting and yelling to the lowest intensities of sleep.

Though caregivers’ and infants’ physiology, emotions, and behaviors have always been triggered by social and contextual stressors, the COVID-19 pandemic provides a clear example of this influence. As we write this piece, almost every family in the United States is navigating the impacts and disruptions of the pandemic on daily life. Specifically, in response to the pandemic, the vast majority of states in the United States enacted some degree of a “shelter in place” mandate and forced social distancing, schools and daycares across the country closed, and the number of unemployed workers in the U.S. labor market skyrocketed (Kochhar, 2020; Lee, Mervosh, Avila, Harvey, & Leeds Matthews, 2020; Mervosh, Lu, & Swales, 2020). These forced disruptions in everyday life illuminate the myriad of ways school, work, income, social connection, and other social factors shape family behaviors, organization, and interactions. Attempting to interpret caregivers’ and infants’ current co-regulatory experiences without placing them within the current context of the pandemic would lead to inaccurate interpretations of physiology, emotions, and behaviors. For example, economic hardship stresses parents and increases the risk of harsh parenting strategies (Conger et al., 2002), and thus, unhealthy patterns of children’s stress physiology which may influence their behavior (Brown, Holochwost, Laurenceau, Garnett, & Anderson, 2021). Observed disruptions in co-regulation and increases in child externalizing behaviors might be misinterpreted as static dysfunctional parenting, or temperamental differences, if not attributed to the context of the COVID-19 pandemic (Ananat & Gassman-Pines, 2020). If research misrepresents the level at which challenges to caregiver-infant interactions and thus co-regulation exists, then researchers will be ill-equipped to design appropriate interventions. The COVID-19 pandemic is an extreme example of how the global, local, social, and economic contexts can affect people’s physiology, emotions, and behavior. Importantly, contextual factors have continuously impacted family life and co-regulation both before and after the pandemic. Accurate understanding of caregiver-infant co-regulation must include assessments of day-to-day contextual influences.

#### 2.1.2. Family culture

Though the co-regulation process is shared among humans, how caregivers co-regulate will be different based on sociocultural experiences. Culture affects the psychological processes affecting relationships, such as how caregivers perceive their relationships and their expectations of their children (Bornstein, 2015; Campos & Kim, 2017). These psychological processes and resulting caregiving behaviors are socialized through cultural norms and caregivers’ early experiences (Sameroff, 2009). Based on their location in the world, social positioning, and customs, caregivers differ in the amount of gaze, vocalizations, and bodily contact behavior they express toward their infants and infants vary in their proximity to their caregiver, physical contact, and interactions with other adults (Feldman, 2007b; Posada et al., 2013). A cross-cultural observational study of mother-infant interactions in their homes showed that mothers in different countries varied in the ways they nurtured, socialized, taught, and spoke to their infants, in addition to the types of materials they used and physical ways they interacted with their infants (Bornstein, 2015). These caregiving behaviors shape infant behaviors, emotion, and arousal. For example, a longitudinal observation study of Nso, West African immigrant, and Italian families by Lavelli, Carra, Rossi, and Keller (2019) found that, among Nso caregiver-infant dyads, motor stimulation (i.e., holding their babies up and down and rocking them back and forth) and rhythmic vocalizing increased infants’ emotional closeness with mothers and their arousal and active attention to the surrounding environment. In West African immigrant dyads, the presence of maternal affectionate talking was associated with a positive feedback loop of increases in infants’ gazing at their mothers’ face and more maternal affectionate talking, as early as four weeks. The infant was oriented toward the mother during bodily contact, which increased the possibility of mutual gaze and longer periods of emotional co-regulation and sharing of positive affect. Cross-cultural comparisons showed the middle-class Italian mothers providing infants with significantly greater proportions of affectionate talking and visual contact for face-to-face communication than Nso and West African immigrant mothers. The presence of affectionate talking and visual contact shaped the foundation for emotional co-regulation of positive emotions, which was characterized by infant smiling and maternal mirroring of infant smiles. Culturally embedded factors influence the ways in which caregivers express, discuss, think about, and respond to their children’s emotions (Raval & Walker, 2019). This process of emotion socialization is also impacted by the caregiver’s preferences for communication, as well as their beliefs about emotion and which emotional qualities are considered desirable in the cultural context that they are raising their infants in (Raval & Walker, 2019). Important for understanding co-regulation, culture guides caregiving behaviors that have been linked to differences in infant physiology, behavior, and emotions.

Caregivers and infants exist within social systems at multiple levels including family, community, city, country, and global contexts (Bronfenbrenner, 1977). In the United States, caregivers socialize their infants within a broader social system of institutionalized power and oppression, where people are stratified and marginalized based on intersecting social categories such as race, ethnicity, gender, sexuality, immigration, and socioeconomic status (SES; Causadias & Umaña-Taylor, 2018; Crenshaw, 1989; Collins, 1986; hooks, 2000). Marginalization is systematically directed toward certain groups (i.e., people of color, women, people with low social class, transgender, gender fluid, and non-heterosexual identities), and this marginalization increases the likelihood of negative physical and mental outcomes and decreases the probability of positive outcomes (Causadias & Umaña-Taylor, 2018; Meyer & Frost, 2013). In the words of Patricia Hill Collins (2016), “whereas the significance of race and class in shaping the context in which motherhood occurs is virtually invisible when white, middle-class women’s experiences are the theoretical norm, the effects of race and class stand out in stark relief when women of color are accorded theoretical primacy” (p. 385). For example, one study showed that, on days when Latinx mothers and fathers experienced acts of discrimination in their workplace, they reported feeling sadder, angrier, and more anxious, and also perceived more problems in their young children’s behaviors (Gassman-Pines, 2015). Belonging to a marginalized racial/ethnic group is associated with higher child cortisol upon arrival to daycare and less rapid declines in cortisol from arrival to midmorning (Brown et al., 2021).

At the same time, families benefit from community cultural wealth, reaping rewards in mental and physical health (Yosso, 2005). Ethnic pride, feeling strong and positive identification with one’s ethnic group, has proven to be a resilience factor, especially for caregivers of color (Collins, 2016; Iturbide, Raffaelli, & Carlo, 2009; Luis Sanchez, Urbina, & D’Anna-Hernandez, 2020; Umaña-Taylor, Updegraff, & Gonzales-Backen, 2011). Experiencing social support promotes supportive caregiving behaviors and better mental health through its reduction of the amount of stress and depression that caregivers feel (Lyons, Henly, & Schuerman, 2005). Thus, caregiver-infant interactions can be conceptualized as reflecting an “adaptive culture - a mix of history, traditions, and adaptive responses to present contextual demands - and not solely as individual patterns of interactions” (García Coll et al., 1996, pg. 1908; Perez-Brena, Rivas-Drake, Toomey, & Umaña-Taylor, 2018).

#### 2.1.3. Family engagement in research

Though infants’ and caregivers’ co-regulatory systems are both shaped by day-to-day experiences and respond to context, much of the existing research has been conducted out of context, both physically (i.e., in research laboratories) and/or theoretically (i.e., ignoring social factors). In other words, co-regulation is rarely assessed as it transpires in the real world (Shiffman, Stone, & Hufford, 2008). Observational and laboratory-based studies have been crucially important for the foundational knowledge of co-regulation. New research can build on this knowledge by asking meaningful questions about the co-regulation process as it occurs and where it takes place. Research aimed at studying caregiver-infant interactions and how their biological, emotional, and behavioral systems co-regulate requires methods that are sensitive to daily fluctuations and can be implemented in home contexts. Understanding naturalistic interactions is possible through methodological advances in scientific research that capture the daily, contextualized processes of physiological, emotional, and behavioral co-regulation. Caregivers can be trained by researchers to describe their experiences and behavior and collect physiological and sleep/activity data from themselves and their infants repeatedly, throughout the course of several days, during their everyday lives. These naturalistic methods safeguard against the generalization that occurs with laboratory-based studies and most self-report measures and provide a more ecologically valid account of caregiver-infant co-regulation (Repetti, Robles, Reynolds, & Sears, 2012). This paper will address methods and mechanisms that translate a contextually-grounded understanding of co-regulation into research design (Table 1).

Though co-regulation occurs in all contexts, it may look qualitatively different based on the cultural and socioeconomic context. Given that culture and family level factors are affecting caregiver-infant co-regulation, researchers need to know the communities that they are studying and tailor the design of the study to fit the lifestyles of the participating families. This can be done by taking a community-based participatory research (CBPR) approach, which is centered on collaborating with caregivers throughout the research project (Israel et al., 2012; Pizarro, Nkosi, & Rios-Cervantes, 2019; Wilson, 2008). The goal of CBPR research is to “promote, in different ways, communities’ interests, health, and safety, and promote justice and the well-being of members of the community” (Deeb-Sossa, 2019, pg. 5). CBPR acknowledges that caregivers are the experts of their experiences and that trusting the voices and insights of the caregivers within the community is essential to fully understanding co-regulation in context (Pizarro et al., 2019). In co-regulation research, caregivers can be asked “to share their knowledge with us in order to culturally adapt best practices” for designing the questions, recruitment, retention, analysis, interpretation, and dissemination (Manzo, Brazil-Cruz, Flores, & Rivera-Lopez, 2020, pg. 52). This is especially important for marginalized caregivers and young children living in low-income environments in the U. S., as researchers have identified a need for tools that appropriately assess parenting across diverse groups that have been oppressed (Causadias & Umaña-Taylor, 2018; Comfort, Gordon, & Naples, 2011; McWayne, Mattis, Green Wright, Limlingan, & Harris, 2017). Researchers should consider taking active efforts to learn the history of the place where they are conducting research, partnering with community members to identify the community’s needs, and supporting collaborating caregivers through research and sustainable interventions (Chilisa, 2020; Israel et al., 2012; Pizarro et al., 2019; Seaton, 2020).

These are introductory guidelines for a humanizing epistemology that fosters a reciprocal relationship between researchers and caregivers (Delgado Bernal, Alemán, Morales, & Mendoza Aviña, 2019). We aim to build a bridge between developmental science and lessons from feminist research and ethnic studies so that the study of caregiver-infant co-regulation can be more inclusive, ethical, and successful in redressing power dynamics, especially when working with marginalized families (Causadias & Umaña-Taylor, 2018). Taking this approach to the study of caregiver-infant co-regulation will ensure the research is asking the appropriate questions, being interpreted in a culturally relevant way, and used to advance social justice by transforming conceptual frameworks or informing culturally grounded policy and programs with the ultimate goal of creating nurturing environments and empowering caregivers to raise happy, healthy babies.

## 3. Studying the daily process of co-regulation in the home

Imagine a day and night in the life of caregivers and infants. The infants cry in the morning, waking up the caregivers. Caregivers experience physiological arousal to meet the infants’ hunger and comfort demands. Caregivers also have competing demands such as feeding themselves and the rest of the family, getting ready for work, and responding to current events. Caregivers experience physiology, emotions, and behaviors triggered by their relationships and their to-do lists, as well as their interactions with their emotionally and physiologically labile infants. For example, a six-month-old wakes one to three times per night, sleeps every 2.5 h during the day, changes their emotional expression once every nine seconds (Malatesta & Haviland, 1982), spends as much as an hour a day crying (James-Roberts & Halil, 1991), and shows a high degree of physiological variation to these emotional expressions (Keenan, Jacob, Grace, & Gunthorpe, 2009). Likewise, caregiver mood might differ based on time of day, work schedules and partner support can change from day to day, and food availability might fluctuate across the month. To understand co-regulation as it happens in daily life, investigators can utilize methodologies with the ability to dynamically assess fluctuating emotions, behaviors, and physiology across the day and night.

Caregivers’ and infants’ lives are comprised of a collection of moments that make up their relationship dynamics and developmental trajectories. Thus, methods that examine the temporal order and dynamics of everyday lives in naturalistic settings are well-suited for the study of caregiver-infant co-regulation (Russell & Gajos, 2020). For example, ecological momentary assessments (EMA) are questions that can be asked repeatedly throughout the day on mobile devices. Salivary bioscience and actigraphy can be used by caregivers in their home settings to assess physiology and rest. These flexible tools enable subjective and objective assessments of caregivers’ own experiences over the course of multiple days, remove barriers to their participation, and can be adapted to fit their schedules and features of their daily lives.

A critical step in understanding caregivers’ and infants’ daily experiences, rhythms, and routines is holding focus groups and qualitative interviews to learn the family and cultural factors that may impact co-regulation. This information will help determine which emotions, parenting behaviors, and sociocultural items need to be included in the research design to assess caregiver-infant interactions. For example, if we know from interviewing and building relationships with parents that they live in multigenerational homes with their children and multiple caregivers, we can include fathers, grandparents, and extended family in the study and/or we can ask about how the moods and behaviors of other family members are affecting the emotions, physiology, and behavior of primary caregivers and infants (Cabrera, Hennigar, Yumiseva-Lackenbacher, & Galindo, 2019; Repetti et al., 2012). If caregivers are working multiple jobs, shifts, or different hours, we can ask about their schedules and match the timing of the questionnaires and physiological samples to their typical schedules. For example, many Mexican heritage women in a study recently conducted by the authors wake up very early with their partners, make them breakfast, and then go back to sleep once their partners leave for work. This daily routine is culturally specific, and with this information, researchers working with Mexican families can adapt the research design and timing of the questionnaires and physiological samples to meet the families’ schedules. Importantly, the interviews can illuminate specific behaviors that caregivers may engage in with their children throughout the day. McWayne et al. (2017) used this method in their work with ethnically diverse, urban-residing, low-income Black mothers to identify positive parenting practices and develop a strength-based, culture- and context-specific questionnaire.

Contextualized co-regulation research can tap into the process as it occurs naturally, assessing physiology and emotions when caregivers and infants wake up in the morning and throughout the day. Caregivers collect emotional, behavioral, and physiological data as they interact with their infants in culturally specific ways, affected by daily stressors and supports, and adapting to the demands of their context. Caregivers can collect emotional, behavioral, and physiological data during caregiver-infant interactions at bedtime and sleep quality throughout the night. Incorporating the behavioral, socioemotional, and physiological together enhances our understanding of day-to-day caregiver-infant interactions (Repetti, Robles, & Reynolds, 2011). Methodological advances provide measures which are sensitive to how routines of daily life (e.g., social rhythms, daily activities, involvement of other family members, daily hassles and stressor) impact emotion, behavior, sleep, and physiology and how they interconnect (Repetti et al., 2012).

Collecting all these data will allow for the exploration of questions like, how do caregivers’ and/or infants’ physiology in the morning affect their emotions and behavior later that day? Similarly, how does the quality of caregiver and/or infant sleep influence their emotions or perceptions of the child’s emotions and behaviors? On days when caregivers express more positive emotion, do they engage in more sensitively responsive ways with their infants and do infants also express more positive emotion? Do infants show healthy patterns of diurnal cortisol when their caregivers respond to their negative emotions with supportive caregiving behaviors? What types of behaviors are supportive for children in which contexts? Do caregivers’ in-the-moment responses impact infants’ wellbeing in the moment above and beyond the overall quality of caregiver-infant interactions? Which contextual daily stressors and supports are most relevant for inhibiting or promoting positive caregiver-infant interactions and co-regulation? In other words, these naturalistic methods allow researchers to pose meaningful questions assessing how co-regulation as experienced by the dyad, the natural variation in these experiences, and the longer-term impacts of those experiences.

These methods are appropriate for addressing questions about caregiver-infant interactions and the essential process of coregulation within naturalistic environments in dynamic, culturally diverse contexts. However, these home-based, in-real-time measures are not intrinsically culturally sensitive or anti-racist. Without consciously incorporating these values into the research procedures, the methods can be used to gain access to extract information from caregivers’ and infants’ daily lives and perpetuate power hierarchies between the researcher and researched (Jayaratne & Stewart, 2008). To achieve the goals of decentering Whiteness and advancing social justice, developmental scientists need to pair these methods with feminist, community-based participatory approaches (Jayaratne & Stewart, 2008). Including the community in the design of the questions as well as the interpretation of the results ensures that the expertise of the caregivers is central in the research (Pizarro et al., 2019). Prioritizing building relationships with caregivers, cultivating trust, and showing respect for their involvement in the science are paramount to this methodology (Manzo et al., 2020). It is not the tools, but the ways the tools are used in relation with others that allows for a deeper examination into this dynamic process of co-regulation that occurs within the context of caregivers’ and infants’ daily lives.

### 3.1. Assessing dynamic fluctuations in emotion and behavior through ecological momentary assessments

Current or recent subjective experiences (i.e., moods, thoughts, behaviors, events) can be assessed using tools like EMA and daily diaries that repeatedly prompt caregivers to describe and share their experiences on a daily, hourly, or momentary basis (Shiffman et al., 2008). Understanding caregivers’ beliefs, thoughts, and feelings may be captured most accurately through self-reported methods that document the individuals’ subjective cognitions and perceptions (Repetti, Wang, & Sears, 2013). Research can investigate caregivers’ perceptions of their own emotions, infants’ emotions, and specific caregiving and infant behaviors at multiple time points (Repetti et al., 2015). For example, caregivers can be asked how happy, sad, or anxious they are feeling and how happy or sad their infants are feeling. Caregivers can also be asked how much of the last 30 min have the infants spent crying or laughing or if they have affectionately touched (i.e., held, caressed, hugged) their infant in the last 30 min (Botero, Langley, & Venta, 2020; Feldman, 2007a, 2007b). In this way, EMA are capturing data on emotional co-regulation in real time (Shiffman et al., 2008) as these emotions and behaviors occur in the naturalistic environment (Repetti et al., 2013). Then, these data can be analyzed to understand links between caregiver and infant, as well as between-dyad and within-dyad differences.

EMA can also be used to collect data on contextual factors to assess within-family predictors of variability in caregiver-infant coregulation across timepoints and test moderation based on more stable individual or family level characteristics (Repetti et al., 2015). For example, caregivers can respond about daily hassles and uplifts, such as whether or not they have argued with their romantic partner or co-parent, felt stressed at work, experienced microaggressions or discrimination, or called someone for social support (Kanner, Coyne, Schaefer, & Lazarus, 1981; Sue et al., 2007). Assessments such as these allow researchers to examine if, when, how, and how much context influences caregiver behavior and co-regulation. Though a caregiver and child might seem highly attuned in the lab, their home context might consistently (or sporadically) interfere with this attunement. An EMA study would illuminate what contextual features seem to most potently interfere, how interference influences caregiver behavior and co-regulation, or what family features effectively block interference. With this design, researchers could answer questions surrounding if co-regulation is disrupted on days when a caregiver experiences microaggressions and if partner support buffers this interference. EMA can also prompt open-ended questions aimed at listening to caregivers’ experiences about the events, identities, emotions, and behaviors that are most salient to them in that moment. They can be encouraged to respond to questions in the language of their choice. Through these responses, researchers and caregivers can collaborate on gaining deeper insights into the process of caregiver-infant co-regulation from the perspective of the caregiver. For example, caregivers can be asked to describe a recent interaction when they felt close, connected, and synchronized with their infant or describe a moment when they felt they did not know how to handle or respond to their infant. Then, these data can be included in mixed-methods studies or analyzed for themes among diverse caregiver-infant experiences in the home.

EMA allow researchers to more accurately tap into the dynamics of the infant and the caregiver as they actually occur in real life. Traditional self-report questionnaires rely on autobiographical memory and summaries of perceptions, emotions, and behaviors that exacerbate measurement error (Repetti et al., 2015). However, EMA are attuned to micro-processes in ways that other methods of data collection are not. They assess the nuances of caregiver-infant interactions in the moment, which is the timescale through which infants and relationships develop (Demirci & Bogen, 2017; Heron, Miadich, Everhart, & Smyth, 2019). This is largely because EMA can be programmed into mobile smartphones and tablets and can be accessed in the home (Heron, Everhart, McHale, & Smyth, 2017). The mobility of these electronic devices and adaptability of this measurement tool into the home increases validity in measuring fluctuating caregiver-infant co-regulatory systems. This especially true because of date- and timestamps on each data entry (Heron et al., 2017).

Researchers should select devices and survey software platforms that caregivers can access freely and use easily and should ask caregivers if they prefer to use their own phones or phones provided by researchers (Smyth & Heron, 2014). Further, questionnaires can be programmed to branch questions based on previous responses, which allows for deeper investigation and also reduces collaborating caregivers’ time burden, since people do not have to respond to questions that do not apply to them (Repetti et al., 2015). The moments when caregivers are prompted to answer questionnaires should be strategically selected based on particular times of interest (i.e., wake, evening, and bedtime), random sampling, or caregiver initiated assessments in response to specific events, such as caregiver-infant interactions (Shiffman et al., 2008; Smyth & Heron, 2014) or infant distress. Multiple measurement schedules can be combined within a single study (Smyth & Heron, 2014). Importantly, caregivers should complete multiple assessments over time to illustrate how co-regulation varies over time and across situations (Shiffman et al., 2008). These qualities increase the ecological validity of EMA and allows researchers to generalize more accurately to caregivers’ and infants’ everyday lives, in addition to examining within-person processes and temporal dynamics (Shiffman et al., 2008; Smyth & Heron, 2014).

### 3.2. Assessing dynamic fluctuations in physiology through salivary bioscience

Caregivers’ and children’s stress physiology are sensitive to daily events, such as caregiver work schedules (Hibel et al., 2014), quality of sleep (Kellerman, Abel, Chong, & Schwichtenberg, 2019), and interactions with other family members (Gordon et al., 2010; Jewell et al., 2015). Salivary markers provide an objective window into infant’s and caregiver’s biological functioning (e.g., brains, genetics, immune system) on a daily basis. Methodological advances in salivary bioscience enable researchers to test a variety of salivary analytes, including measures of hormones, cytokines, immunoglobulins, enzymes, DNA, environmental chemicals, elements, and metals (Granger & Taylor, 2020). One of the most prominent biomarkers of caregiver-infant co-regulation is cortisol, which helps researchers understand physiological responses to social and psychological stressors and the role of caregivers as a buffer and conduit for children’s stress (Gunnar, 2020). Another biomarker, salivary alpha amylase, is a potential surrogate marker of the autonomic nervous system; however, the evidence is mixed and is not as robust as cortisol (Hibel et al., 2020). Saliva can be collected throughout the day at multiple time points providing diurnal changes in caregivers’ and infants’ physiology (Hibel et al., 2020; Ramirez et al., 2017) or at time points that are physiologically salient depending on the particular analyte(s) of interest (Hernández & Taylor, 2020). Researchers have identified clear protocols for in-home implementation and caregivers can be trained on these protocols (Padilla, Calvi, Taylor, & Granger, 2020). The analyte(s) being detected will dictate where in the mouth to sample (e.g., under the tongue, in cheek pocket; Hernández & Taylor, 2020) and the timing of collection (Padilla et al., 2020). Salivary bioscience allows for a relatively low-cost, minimally invasive assessment of infant-caregiver biological functioning in response to naturally occurring interactions in the dyad’s typical contexts (Halpern, Whitsel, Wagner, & Harris, 2012; Walia & Mehra, 2019).

For salivary bioscience methods to accurately capture how caregiver-infant physiology is co-regulating throughout the day, caregivers must be compliant with timelines and behavioral restrictions. Strategies for enhancing compliance include clearly explaining the importance of the research, outlining the protocol for collection timing and handling, practicing with the caregiver, answering any questions, providing detailed instructions and research team’s contact information, providing all supplies in packages for ease of collection, and contacting caregivers at or before sample collections with reminders (Padilla et al., 2020; Stalder et al., 2016). Researchers can encourage and support caregivers in successfully complying to the protocol through in-person instructions of methods, phone call and/or text reminders, and color-coding supplies to ensure that all caregiver supplies are the same color and infant sample supplies are a different color (Laudenslager, Calderone, Philips, Natvig, & Carlson, 2013; Valentino, De Alba, Hibel, Fondren, & McDonnell, 2017). Collecting saliva samples from infants can be challenging, depending on the age and temperament of the infant. Distraction via engaging light-up toys can assist with saliva collection from fussy infants. Further, caregivers are instructed to store samples in the freezer. If their socioeconomic status limits caregivers’ access to a consistent electrical power source for sample freezing, researchers should provide a cooler or another cold storage option (Padilla et al., 2020). Caregivers should report medication use (including hormone-based contraceptives), document vigorous exercise or acute illness, and limit eating, drinking, and smoking 10–20 min before collections (Padilla et al., 2020). To ensure that particular sampling times are being followed, caregivers should record the collection times in logs or through time-stamped photographs following each sample (Padilla et al., 2020). This assures that the daily physiological data collected through salivary bioscience methods is adequately representing caregiver-infant co-regulation.

Neurobiological systems are dynamic and responsive to their environments, and there is still much to learn about how caregivers’ and infants’ physiological changes are associated with emotional and behavioral co-regulation in context (Gunnar, 2020). Collecting saliva across the day in naturalistic contexts has begun to shed light on these processes. For example, living in conditions of higher sociodemographic burden has been associated with infants’ blunted diurnal cortisol slopes, higher caregiver and infant diurnal cortisol output, and reduced caregiver-infant cortisol synchrony (Clearfield, Carter-Rodriguez, Merali, & Shober, 2014; Perrone et al., 2021). For families living in adversity, increases in parental sensitivity are associated with improvements in infants’ stress-induced cortisol regulation (Berlin, Martoccio, Bryce, & Harden, 2019). Parents’ stress spills over to affect their parenting and how parents respond during these times of challenge is thought to be more critical in determining child regulation than parenting in non-distress contexts (Bowlby, 1969; Malia, 2007; Rutherford, Wallace, Laurent, & Mayes, 2015). Incorporating dynamic biobehavioral assessment in naturalistic studies allows researchers to answer these questions. Emotional and physiological distress can be difficult to provoke in the lab (Gunnar, Talge, & Herrera, 2009), and may not be representative of typical maternal responses (Repetti et al., 2012). Repeated momentary measures could capture infant distress, physiological reactivity, maternal responses, and dyadic co-regulation as it naturally occurs.

Calculating diurnal hormonal rhythms (i.e., levels and slopes) is most accurate with at least three saliva collections across the day at morning, midday, and bedtime (Smyth, Hucklebridge, Thorn, Evans, & Clow, 2013). Multiple days of saliva sampling is recommended to understand how caregivers’ and infants’ hormones vary across the day and distinguish between trait and state level differences (Hellhammer et al., 2007). Once the salivary data is collected and assayed, there are multiple methods of analyzing diurnal rhythms and slopes (see Pruessner, Kirschbaum, Meinlschmid, & Hellhammer, 2003), as well as caregiver-infant physiological synchrony (see Helm, Miller, Kahle, Troxel, & Hastings, 2018). Longitudinal studies collecting hormones at numerous time points in home settings from caregivers and infants are rare. A longitudinal study found that flatter diurnal cortisol slopes and more positive cortisol awakening responses at 14 months were associated with shorter nighttime sleep duration between 14 and 36 months (Saridjan et al., 2017). Incorporating multiple saliva collections surrounding bedtime and naptime could uncover how caregivers co-regulate infants’ physiological arousal and help infants establish adult-like sleep patterns. Integrating multiple systems provides new perspectives on the impact of hormones on physiological and behavioral processes in different cultural contexts and childrearing conditions across this important developmental period (Feldman & Bakermans-Kranenburg, 2017). Assessing naturalistic caregiver-infant interactions allows for a scientific understanding of caregiver-infant co-regulation in response to the challenges they face on a daily basis and therefore provides evidence of momentary fluctuations in co-regulation and the subsequent development of biological and emotion regulation.

### 3.3. Assessing dynamic fluctuations in rest and movement through actigraphy

Integrating moment-to-moment movement and sleep data allows for an understanding of caregiver-infant co-regulation that extends from day to night. Caregivers’ activity rhythms shape infants’ rhythms and synchronize them with circadian patterns of light and dark (Tsai, Barnard, Lentz, & Thomas, 2011). Sleep behaviors are embedded in the sociocultural context and actigraphy methods have been developed to measure sleep in the family home environment (Owens, 2005; Sadeh, 2011; Vélez-Agosto et al., 2017).

Actigraphy watches are non-invasive monitoring devices that can be worn 24 h a day or nightly to measure caregiver and infant movements, and thus sleep and wake patterns, in the home context (Buxton, Nahmod, & Strayer, 2017; Martin & Hakim, 2011). Caregivers are instructed to wear the actigraph device on their wrists, though infants should wear the devices on their ankles (Meltzer, Montgomery-Downs, Insana, & Walsh, 2012). Using validated computer-based scoring algorithms, actigraphy data are translated into 1-minute epochs that categorize the data into sleep and wake (i.e. movement). Algorithms determine the times of sleep onset and offset, number of night awakenings, the amount of time spent awake after sleep onset, and total sleep time (Meltzer et al., 2012). Actigraph models may have additional abilities to track the distance between caregivers and infants, which can aid in understanding co-regulation throughout the night, especially as it relates to co-sleeping and bed-sharing. Researchers using actigraphy in sleep are advised to report on their methodological choices, including the devices, software, placement, algorithms, epoch length, and scoring rules for coding (see Meltzer et al., 2012).

Actigraphy is a valid method for measuring infant sleep, including infants younger than six months old (So, Buckley, Adamson, & Horne, 2005). Actigraphy-recorded sleep in infancy has been highly correlated with parental reports of younger infants’ sleep and less correlated as infants develop (So, Adamson, & Horne, 2007; Tikotzky & Volkovich, 2019). This suggests that caregivers become less aware of infant night awakenings as infants become better able to self-soothe (Tikotzky & Volkovich, 2019). Thus, actigraphy is a useful tool in measuring sleep, above and beyond parental reports. Actigraphy can also be used to measure physical activity in caregivers and infants (Pitchford, Ketcheson, Kwon, & Ulrich, 2017; Wang, Chen, Tung, Lee, & Tsai, 2019). In addition to being a valid measure of sleep and activity, the strengths of actigraphy include being easy to use, cost-effective, and representative of real life in its ability to be used at home during multiple days, though it should be noted that actigraphy may not always accurately distinguish between periods of sleep and sedentary behavior (Buxton et al., 2017). Therefore, caregiver reported EMA should be used to provide subjective accounts of sleep and rise times to supplement the actigraphy data and accurately determine the sleep periods and movement periods throughout the day and night (Sadeh, 2011; Sadeh & Acebo, 2002). Furthermore, to ensure more reliable data, caregivers should be instructed to press the event marker on the actigraphy watches when caregivers and infants fall asleep and wake up (Martin & Hakim, 2011). Using the subjective reports from EMA and event markers on the devices as guides ensures the most accurate scoring of the actigraphy data (see Walia & Mehra, 2019).

## 4. Supporting and collaborating with caregivers throughout the research process

Dynamically assessing emotions, behaviors, physiology, and sleep within naturalistic contexts requires caregivers to work as collaborators in the research as they collect saliva from themselves and their infants, wear the actigraphy watches, and/or answer questionnaires repeatedly throughout the day in their homes. The process is intensive and potentially invasive, asking a lot of the participating families. Collecting data in the home necessarily causes an interruption in ongoing activity in the caregivers’ and infants’ daily lives (Barta, Tennen, & Litt, 2012). Caregivers collaborating in research studies must alter, though minimally, their typical routines to include collecting saliva, answering EMA questionnaires, and pressing buttons on the actigraph (Repetti et al., 2015). This participant burden, loss of privacy during in-home visits, and potential for abuse and exploitation of biological samples among Black, Indigenous, people of color (BIPOC) may be overwhelming and alienating for caregivers, who may be skeptical about participating in research. Importantly, if caregivers refuse or hesitate to use home-based repeated sampling methods like EMA, salivary bioscience, and/or actigraphy, researchers should not pressure them and instead aim to understand why these methods are alienating. Alternatively, when multiple methods are used, researchers can allow participants to self-select the measures that most comfortably fit their values and schedule. For example, for families who are apprehensive to provide saliva, EMA and sleep data can be collected and still provide important information regarding co-regulation in that family. Further, not all research questions necessitate the collection of all of these assessments, thus not every naturalistic study of co-regulation must include EMA, saliva, and actigraphy. Importantly, the research process from start to finish must strive to achieve ethical validity. That is, “methods are only ‘valid’ if they align with and emerge from the ethical principles that guide the communities in which we work (which are linked to both the ways of being and knowing in these communities),” (Pizarro et al., 2019, pg. 47).

There is a power dynamic at play when caregivers and infants are being assessed by research assistants (Wolf, 1996). This power dynamic may be especially pronounced when Black, Latinx, Asian, and/or Indigenous caregivers and infants are assessed by White research assistants from primarily White institutions. This power dynamic may influence caregivers to engage in more socially desirable behaviors or be careful and alert in the researchers’ presence. This is partly why engaging with the community, building trust, and collaboration are so critical. In establishing trust with the research team, participants will share more about the challenges and joys of caregiving, be more honest and thorough in their responses, allowing the research to represent their experiences more accurately. The research team must value and support caregivers throughout the process, and the caregivers must feel valued and supported by the research team (Chilisa, 2020; Pizarro et al., 2019; Wilson, 2008).

When researchers’ cultural intuition matches the caregivers’, it facilitates the relationship building process and creates a sense of cultural safety (Manzo et al., 2020). Relationship building is at the core of cultural safety, which gives power to community members to say whether or not they feel safe and the responsibility to researchers to create genuinely trusting, safe spaces (Israel et al., 2012; McCubbin, 2006). The relationship quality is strengthened by researchers’ willingness and effort to support the caregivers, above and beyond their participation in the research study (Manzo et al., 2020). It is recommended to be consistent and engaged with caregivers, understanding of their needs, and active in helping them overcome their difficulties (Manzo et al., 2020). Partnering with caregivers and community, co-learning, and attending to social inequalities is a rewarding, time-consuming process that requires care and commitment (e.g., Israel et al., 2012).

It is recommended to approach the work with cultural humility and build empowering and power-sharing partnerships with the caregivers who are collaborating in the work (see Israel et al., 2012). Cultural humility refers to a constructive process of self-critique and awareness, which includes a constant questioning of biases, assumptions, and the researcher’s role as ‘objective’ or ‘neutral’, along with active efforts to address power imbalances between researcher and participant (Delgado Bernal, 1998; Freire, 2018; Tervalon & Murray-Garcia, 1998). Researchers are encouraged to disrupt academic norms requiring scholars to be disembodied and impersonal (Delgado Bernal et al., 2019). Instead, they should be fully present and bring their whole selves, their “bodymindspirit,” to their relationships with caregivers and families (Delgado Bernal et al., 2019; Lara, 2002). It is also the hope that researchers utilize their resources, power, and data to promote more equitable societies for caregivers to nurture their infants in (Israel et al., 2012).

Working in partnership with caregivers and infants requires flexibility. Families’ schedules may be busy and unpredictable, requiring rescheduling at the last minute or working on weekends. Resources must be budgeted for high levels of caregiver involvement, staff training, and coordination (Repetti et al., 2012). Resources must also be mobilized to create focus groups and/or qualitative interviews with the caregivers, build trusting relationships with the community, understand feasibility, and design the details of the study to make sure it encompasses the salient features of their daily lives (Chilisa, 2020). This paper focuses on the U.S. context; however, researchers will have to tailor their designs to the particular communities they are working with and contexts in which they work.

Oftentimes, researchers who study caregiver-infant interactions attempt to control settings (i.e., bringing families into the laboratory and providing toys to play with) and strip families from their cultural context, ignoring and undervaluing the influence of culture. Importantly, the majority of co-regulation research has focused on White samples, using tasks which were validated in predominantly White samples, with caregivers responding to questionnaires developed with a White perspective (e.g., Stifter & Fox, 1990; Tronick, Als, Adamson, Wise, & Brazelton, 1978; Tronick, Mueller, DiCorcia, Hunter, & Snidman, 2020). In other words, there has been an assumption that the perspectives, experiences, and psychological processes of White families are universally applicable (Cundiff, 2012). Critically, if co-regulation research is limited to White families, the knowledge base of the field is actually substantially smaller than assumed, given that the global majority of people are people of color. This paper is aligned with current calls for decentering Whiteness and reckoning with the ways that experiences of Black, Indigenous, Latinx, Asian, and queer caregivers and family structures have been marginalized by dominant institutions, including academia (Roberts, 2020; #ShutDownAcademia, 2020). For women of color, especially, there is a need for personalized, self-defined standpoints on motherhood in social science as there is a disconnect between “what has been said about subordinated groups in the dominant discourse, and what such groups would say about themselves if given the opportunity” (Collins, 2016, pg. 48). By misrepresenting or underrepresenting caregivers and infants identifying with races, ethnicities, classes, genders, and/or sexualities marginalized by dominant structures, research is perpetuating culturally insensitive deficit models and failing to support contextually appropriate adaptive functioning (Cabrera et al., 2019; Causadias & Umaña-Taylor, 2018; Labella, 2018). By including caregivers and infants from marginalized groups and asking caregivers to share about their daily routines and home lives, researchers can include culturally relevant information and ask appropriate questions. Once researchers analyze the data according to culturally relevant research questions based on conversations with community members, they should engage with the caregivers to examine the findings and question the interpretations (Pizarro et al., 2019). It is key to present the data and results to the caregivers who are collaborating in the research (Deeb-Sossa, 2019). This approach will strengthen the ability of scholarly research to document and depict caregiver-infant interactions and co-regulation in a socially just way (Delgado Bernal et al., 2019).

## 5. Conclusion

The study of co-regulation is based on the knowledge of the interconnectedness among caregivers and infants and their developing, fluctuating, and adaptive regulatory systems. It is aligned with developmental theories and philosophies of interbeing, which describe the interconnectedness of individuals with their relational, social, and global contexts (Bronfenbrenner, 1977; Hanh, 1987). This interdependence is especially highlighted in infancy, when infants rely heavily on caregivers to meet their needs for survival. Infancy is a sensitive period for co-regulation, because the early organization of neurobiological systems is crucial for the cognitive, socioemotional, and self-regulatory skills in childhood and adulthood (Bornstein, 1989). Many existing studies of co-regulation assume that the behaviors, physiology, and emotions captured on one day in the laboratory, just a short snapshot of caregiver-infant life, can be generalized to naturalistic settings. This approach misses out on the fluctuations and lability of these dynamic processes. Co-regulation in context involves studying biopsychosocial systems, within a relationship, in the home, while taking into account social, cultural, and economic factors, over the course of multiple days. The methodological advances discussed contribute to greater ecological validity of the dynamic and context-dependent process of co-regulation and support inquiry into naturalistic within-person and within-dyad regulatory processes (Russell & Gajos, 2020). These research methods allow for integration of the study of emotion, behavior, physiology, and sleep for a holistic, contextualized view into caregiver-infant co-regulation. Understanding how caregivers and infants co-regulate in their natural contexts by dynamically assessing emotions, behaviors, and physiology will enable science to test theories of co-regulation in life as it is lived. Further, it will enable the design of interventions to support families in the ways they need to be supported.

We suggest that researchers should consider embracing the complexity of studying co-regulation in diverse cultural contexts and the responsibility to advance social justice with their work (Campos & Kim, 2017; Lerner & Overton, 2008; McCall, 2005). Home-based methods make research more accessible in different cultural contexts, because they do not require families to uproot from their homes and travel to laboratories with different cultural expectations. This paper offers methodological tools to work with caregivers and train them to measure their infants’ and their own physiology, emotion, behaviors, and sleep. It also argues that these methods should be paired with a humanistic, partnership approach. Thus, studies should extend the current literature and include caregivers and infants from various socioeconomic, ethno-racial, and cultural backgrounds to understand the within-group and within-person diversity of experiences in this shared human developmental process. Researchers must be aware and conscious of redressing power dynamics as to not reproduce or perpetuate systems of oppression within their work. This is especially true when working with caregivers and infants who are marginalized due to their social positioning. The benefits of having ecological and ethical validity in research outweigh the costs. Accurate representation is critical for valuing the important work that caregivers contribute to society by raising infants. These findings will provide the evidence-base for designing culturally relevant programs and policies to support caregivers in raising healthy infants within their communities. As Gloria Anzaldúa (2015) wrote, “May we do work that matters. *Vale la pena.”* It’s worth it.

## Figures and Tables

**Table 1:** Assessing Features of Caregiver-Infant Co-regulation.

Features of Caregiver-Infant Co-regulation	Methods
Co-regulation varies within a dyad	
Co-regulation is influenced by momentary stressors and supports	Multiple assessments
Stressors and supports vary across the day	
Culture influences how caregivers behave	
Co-regulation is influenced by household routines/chaos	Home-based measurements
Co-regulation is a biobehavioral process	
Behavior and biology dynamically fluctuate	
Emotions are responsive to the environment and socialized by culture	
Caregiver and infant physiology are influenced by family’s daily experiences and interactions	EMA, salivary bioscience, actigraphy
Sleep is bidirectionally associated with emotions, caregiver-infant relationship quality, and physiology	
Caregivers are the experts of their families’ experiences	Community-based participatory research approach to collaborate in designing relevant studies with ethical and ecological validity
Culture influences how caregivers behave and the features of their daily lives	
Co-regulation fluctuates across naturally occurring events	Timing the assessments at ecologically valid moments in caregiver-infant daily life (i.e., after work, bedtime)
